# Early pneumothorax as a feature of response to crizotinib therapy in a patient with *ALK* rearranged lung adenocarcinoma

**DOI:** 10.1186/1471-2407-13-207

**Published:** 2013-04-26

**Authors:** Spyridon Gennatas, Susana J Stanway, Robert Thomas, Toon Min, Riyaz Shah, Mary ER O’Brien, Sanjay Popat

**Affiliations:** 1 Royal Marsden Hospital, London, UK; 2 Royal Marsden Hospital, Surrey, UK; 3 Kent Oncology Centre, Maidstone and Tunbridge Wells NHS Trust, Kent, UK

**Keywords:** Lung cancer, Lung adenocarcinoma, ALK rearrangement, Pneumothorax, Early pneumothorax, Crizotinib, ALK rearranged lung adenocarcinoma

## Abstract

**Background:**

Single arm phase 1 and 2 studies on Crizotinib in ALK-positive patients so far have shown rapid and durable responses. Spontaneous pneumothoraces as a result of response to anti-cancer therapy are rare in oncology but have been documented in a number of tumour types including lung cancer. This includes cytotoxic chemotherapy as well as molecular targeted agents such as gefitinib and Bevacizumab. These often require chest drain insertion or surgical intervention with associated morbidity and mortality. They have also been associated with response to treatment. This is the first report we are aware of documenting pneumothorax as response to crizotinib therapy.

**Case presentation:**

A 48-year-old Caucasian male presented with a Stage IV, TTF1 positive, EGFR wild-type adenocarcinoma of the lung. He received first line chemotherapy with three cycles of cisplatin-pemetrexed chemotherapy with a differential response, and then second-line erlotinib for two months before further radiological evidence of disease progression. Further analysis of his diagnostic specimen identified an ALK rearrangement by fluorescence in situ hybridization (FISH). He was commenced on crizotinib therapy 250 mg orally twice daily. At his 4-week assessment he had a chest radiograph that identified a large left-sided pneumothorax with disease response evident on the right. Chest CT confirmed a 50% left-sided pneumothorax on a background of overall disease response. A chest tube was inserted with complete resolution of the pneumothorax that did not recur following its removal.

**Conclusion:**

Our case demonstrates this potential complication of crizotinib therapy and we therefore recommend that pneumothorax be considered in patients on crizotinib presenting with high lung metastatic burden and with worsening dyspnoea.

## Background

Crizotinib is approved for *ALK* rearranged relapsed non-small cell lung cancer with most patients treated in the initial expanded cohort phase 1b study acquiring durable responses. The overall response rate was 57% (CI 46-68) and the disease-control rate was 87% [1]. A retrospective study on the same population by Shaw et al. suggested that overall survival for patients with advanced ALK-positive NSCLC was significantly longer in patients given Crizotinib second- or third-line compared to Crizotinib-naïve patients, whose prognosis was very similar to the general NSCLC population. This evidence indicates Crizotinib might prolong overall survival in ALK-positive NSCLC patients [2]. Regarding the timing of responses, in both the phase I and phase II trials, the majority were achieved within 8 weeks of treatment initiation. The duration of response was 48.1 and 41.9 weeks respectively indicating a very rapid and prolonged response. Treatment was generally well tolerated with gastrointestinal (Grade 1/2) and visual disorders (Grade 1) being the commonest [3,4].

## Case presentation

**Figure 1:**
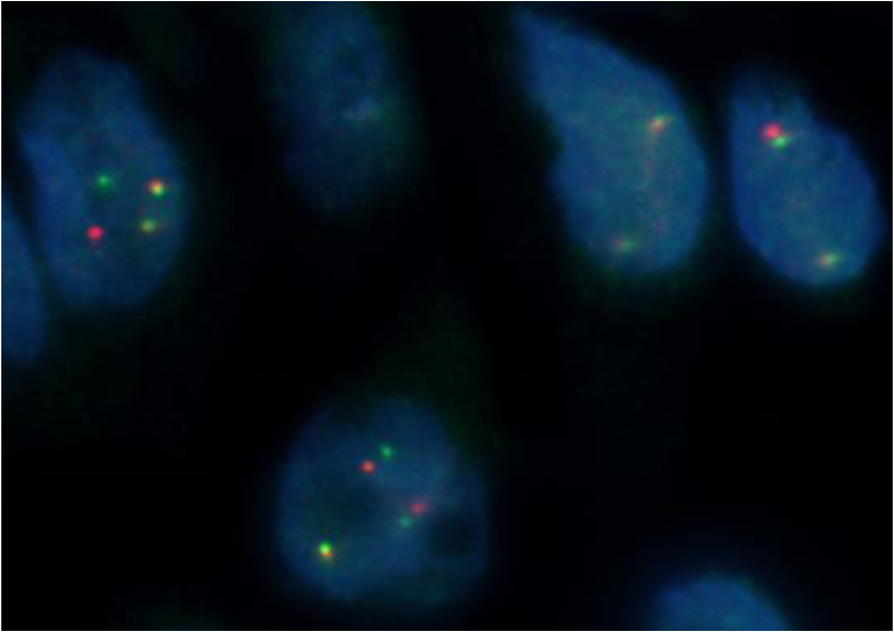
**ALK rearrangement as visualised by fluorescence in situ hybridization (FISH).** FISH image of ALK re-arrangement using the Vysis (Abbott) LSI ALK dual color, break apart rearrangement probe (Abbott molecular, Illinois).

**Figure 2:**
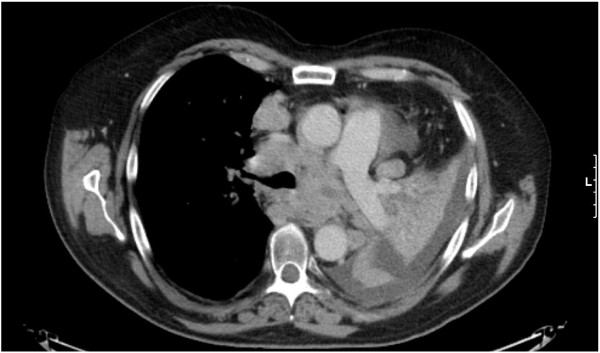
**Baseline staging CT scan.** CT scan of the chest prior to commencing treatment with crizotinib exhibiting extensive metastatic lung disease, mediastinal lymphadenopathy and a partially collapsed left lung.

**Figure 3:**
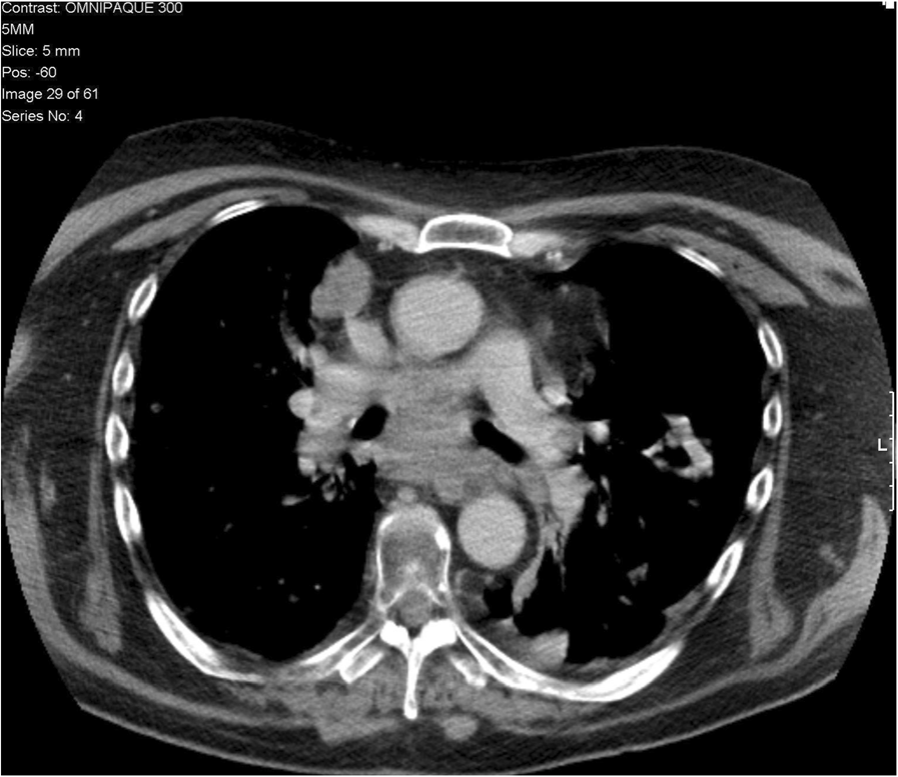
**Chest CT showing disease response.** Staging CT scan after 4 weeks of crizotinib treatment showing disease response with improvement in mediastinal nodal disease, lung metastases, and re-canalization of the left main bronchus.

**Figure 4:**
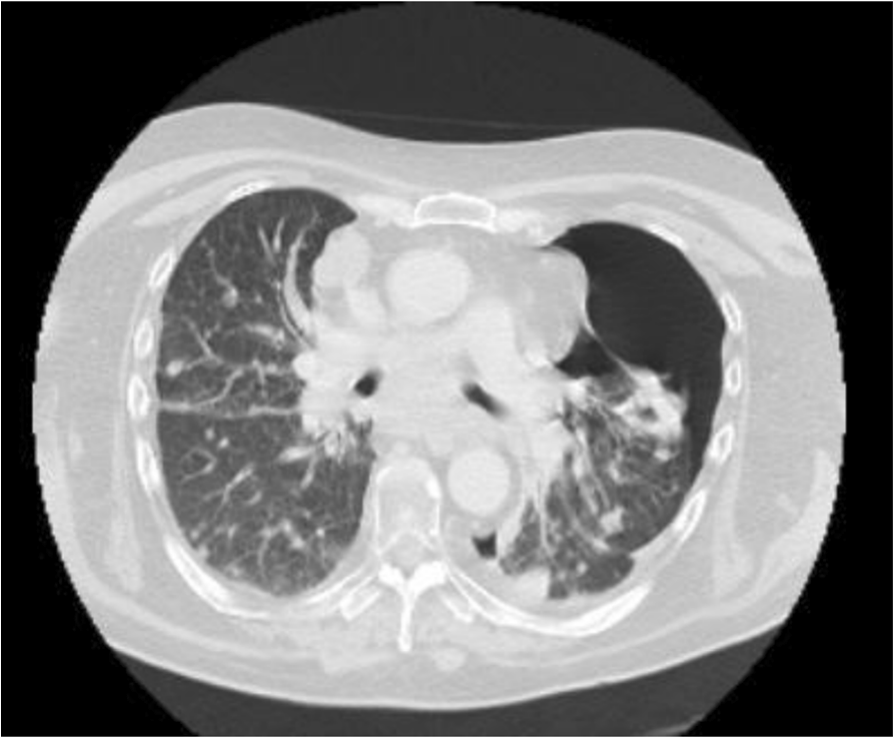
**Chest CT showing disease response and a 50% left-sided pneumothorax.** Staging CT scan following 4 weeks of treatment showing disease response and a 50% left-sided pneumothorax on ‘lung windows’.

**Figure 5:**
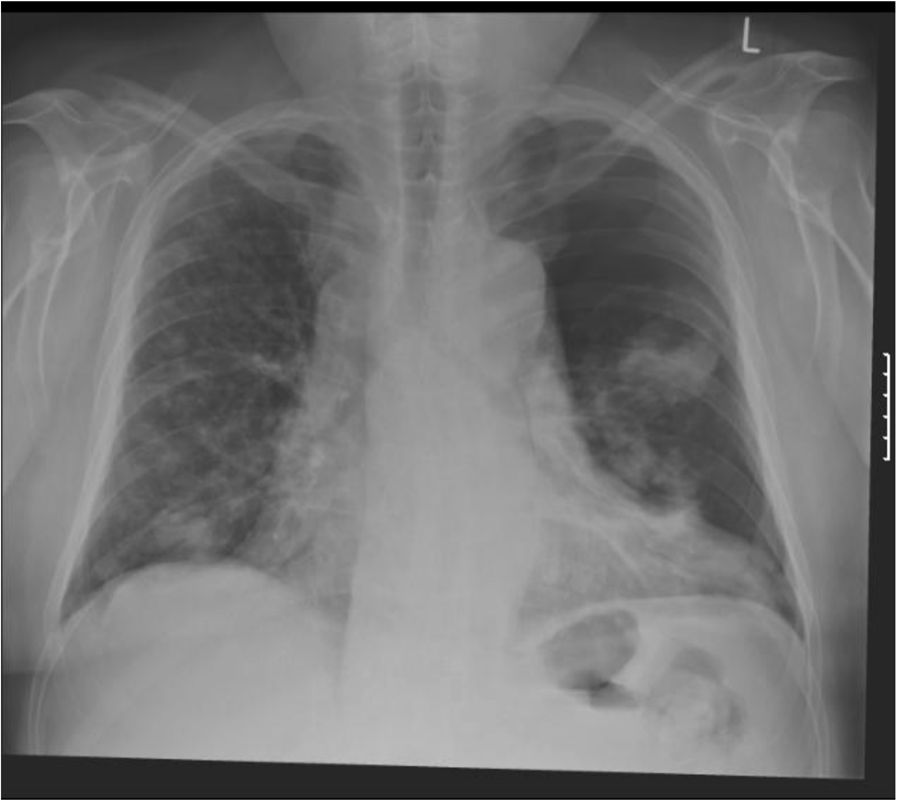
**Chest radiograph showing a 50% left-sided pneumothorax.** 50% left-sided pneumothorax as visualised on a simple chest radiograph following 4 weeks of crizotinib treatment.

**Figure 6:**
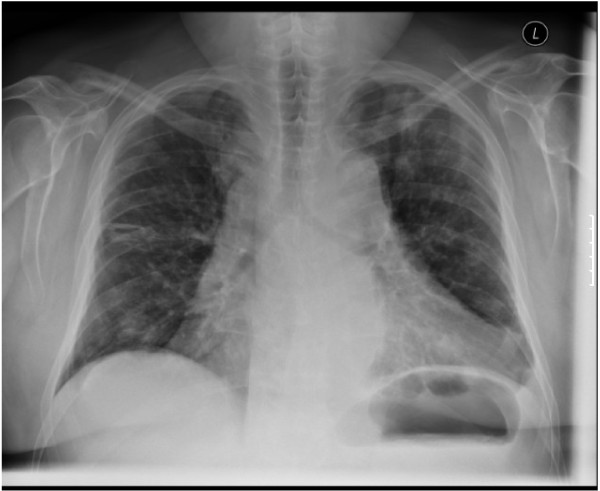
**Chest radiograph following removal of chest drain.** Complete re-expansion of the left lung as seen following removal of left-sided chest drain for spontaneous pneumothorax secondary to response to treatment with crizotinib.

A 48-year-old Caucasian male ex smoker (10 pack-year tobacco exposure) presented in 2011 with cough and back pain. Computerized tomography (CT) imaging showed a lung primary and metastases to both lungs, spine, and left adrenal. A computerized tomography (CT)-guided biopsy confirmed TTF1 positive, EGFR wild-type adenocarcinoma of the lung. He received three cycles of cisplatin-pemetrexed chemotherapy with a differential response, and then second-line erlotinib for two months before further radiological evidence of disease progression. Erlotinib was administered based on the findings of the BR.21 phase III trial, which demonstrated that erlotinib prolonged survival in relapsed NSCLC unselected by EGFR genotype following progression on first- or second-line chemotherapy compared to placebo (6.7 months vs 4.7 months) [5]. He subsequently received radiotherapy to the spine for palliation (8Gy, single fraction) followed by radiotherapy to the mediastinum due to lymphadenopathy (20Gy, 5 fractions). Thereafter, he was referred for further evaluation, where analysis of his diagnostic specimen identified an *ALK* rearrangement by fluorescence *in situ* hybridization (FISH) using the Vysis (Abbott) LSI ALK dual colour, break apart rearrangement probe (Abbott molecular, Illinois, Figure 1). Following a baseline staging CT (Figure 2), which confirmed multiple metastatic sites, including the lungs and adrenal gland, as well as a chronically collapsed left lung (first seen on CT prior to commencing erlotinib), he was commenced on crizotinib therapy 250 mg orally twice-daily. At his 2-week assessment, he reported marginal clinical improvement. At his 4-week assessment he complained of worsening dyspnoea, and a chest radiograph identified a large left-sided pneumothorax with disease response evident on the right. Chest CT confirmed a 50% left-sided pneumothorax on a background of overall disease response (Figures 3 and 4) with improvement in mediastinal nodal disease, lung metastases, and re-canalization of the left main bronchus. The patient was admitted and chest tube was placed with an underwater seal. Crizotinib was continued throughout. The left lung re-expanded fully within 24 hours, with correspondent symptomatic improvement and remained fully expanded after removal of the chest tube (Figure 5 and 6) at which point the patient was discharged. A few weeks later he deteriorated clinically due to likely disease progression, active treatment was discontinued and his care was transferred to the community palliative care team.

## Discussion

Case reports of remarkable responses to Crizotinib are emerging. Kim et al., reported a case of a 14-year old girl with extensive Stage IV (multiple lymph nodes and lymphangitic lung metastasis), ALK-positive lung adenocarcinoma, who was treated with first-line Crizotinib. An FDG-PET at Day 21 revealed an impressive partial response across all disease sites. A 9-week CT revealed a 76.3% decrease in tumour bulk and at 64 weeks the patient remained at confirmed partial response [6].

Spontaneous pneumothoraces as a result of response to anti-cancer therapy are rare in oncology and typically occur in cases of metastatic osteosarcoma or germ cell tumours, with case reports in other histologies eg. breast cancer [7]. Although, spontaneous pneumothoraces have also been described in primary lung cancer (including small cell and NSCLC) as initial presentation or as complication, more commonly, of chest radiotherapy, they have been very rarely associated with cytotoxic chemotherapy [8,9]. A retrospective evaluation at a single centre from Maniwa et al, demonstrated that patients with pneumothorax as a result of treatment for pulmonary malignancy (primary or metastatic) often require prolonged chest tube drainage and sometimes surgical drainage, which is associated with increased peri-operative morbidity and mortality, highlighting the significance of early diagnosis and prompt expert management of such cases [10].

Molecular targeted therapies have also been linked with the development of spontaneous pneumothoraces. Mori et al., reported a case of bilateral spontaneous pneumothoraces within four weeks of initiation of gefitinib treatment in a patient with multiple bilateral pulmonary metastases. These were only small and resolved spontaneously. Interestingly there was associated treatment response in both lungs within the same time period as seen on CT [11]. Yang et al., reported a case of Bevacizumab-induced pneumothorax in a patient treated for metastatic colorectal cancer, including multiple lung metastases. This required chest tube drainage for 5 days with complete resolution of the pneumothorax, highlighting the importance of considering this diagnosis in patients with acute chest discomfort while on Bevacizumab [12]. This is the first report we are aware of documenting pneumothorax as response to crizotinib therapy. Proposed mechanisms include rapid tumour lysis and tissue necrosis in response to cytotoxics leading to rupture into the pleural cavity or bronchopleural fistula development [8]. In our case, imaging did not suggest obvious macroscopic pleural disease with no pleural nodularity evident, although several of his lung metastases did abut the pleura.

## Conclusion

Our case demonstrates that spontaneous pneumothoraces are a potential complication of crizotinib therapy and we therefore recommend that pneumothorax is considered in patients on crizotinib presenting with high lung metastatic burden and with worsening dyspnoea.

### Consent

Verbal informed consent was obtained from the patient on the 24^th^ of April 2012 for publication of this case report and any accompanying images. His family were present at the time. The patient is unfortunately now deceased.

## Competing interests

The authors declare that they have no competing interests.

## Authors’ contributions

TM carried out the fluorescence in situ hybridization (FISH) studies. RS provided Figure 1 and RT provided the radiographic images. SG, SS and SP examined, treated and observed the patient, including follow-up. SG, SS, MO and SP participated in writing the manuscript. All authors read and approved the final manuscript.

## Pre-publication history

The pre-publication history for this paper can be accessed here:

http://www.biomedcentral.com/1471-2407/13/207/prepub
